# Expression of TXNIP is associated with angiogenesis and postoperative relapse of conventional renal cell carcinoma

**DOI:** 10.1038/s41598-021-96220-y

**Published:** 2021-08-25

**Authors:** Mate Meszaros, Maria Yusenko, Lilla Domonkos, Lehel Peterfi, Gyula Kovacs, Daniel Banyai

**Affiliations:** 1 Department of Urology, Medical School, University of Pecs, Pecs, Hungary; 2 Institute of Biochemistry, University of Muenster, Muenster, Germany; 3 Medical Faculty, Ruprecht-Karls-University, Heidelberg, Germany

**Keywords:** Biomarkers, Oncology, Urology

## Abstract

One of the common mediator of tumour progression is the oxidative stress induced by inflammatory tumour microenvironment (TME). Activated fibroblasts, local and immune cells produce reactive oxygen species (ROS) supporting tumour cell proliferation and pave the way for metastatic tumour growth. TXNIP regulates ROS generation by inhibiting the antioxidative function of thioredoxin (TXN). The shift of TXNIP/TXN balance towards overexpression of TXNIP is associated with proliferation of endothelial cells during tumor angiogenesis. The oxidative stress activates the hypoxia inducible factor-1 (HIF-1), which plays an important role in the biology of conventional RCC (cRCC). Under oxydative stress TXNIP interacts with NLRP3 inflammasome leading to maturation and secretion of inflammatory cytokine IL1β. To establish the role of TXNIP and downstream genes HIF1α and IL1β in the biology of cRCC, we have applied immunohistochemistry to multi-tissue arrays containing tumours of 691 patients without detectable metastases at the time of operation. We found that cRCC displaying a fine organised capillary network with nuclear translocation of TXNIP and expressing IL1β have a good prognosis. In contrary, we showed a significant correlation between cytoplasmic TXNIP expression, inefficient vascularisation by unorganized and tortuous vessels causing tumour cell necrosis and postoperative tumour relapse of cRCC.

## Introduction

Conventional renal cell carcinoma (cRCC) makes up 80% of malignant kidney tumours. Approximately 40% of the patients with cRCC have a metastasis at the time of operation or will develop metastatic disease during the postoperative course of 5 years^1^. Introducing modern imaging techniques resulted in a growing number of patients with incidentally detected small renal tumours confined to the kidney^2,3^. However, approximately 15% of clinically localised tumours operated with curative intent develops metastasis within 5 years follow-up. Recent drug therapies can not cure the disease, but they may prolong the life of patients with metastatic disease^4,5^. Therefore, it is a need for biomarkers to identify a group of patients with high risk of postoperative tumor relapse to be able to start adjuvant therapy as early as possible.

The inflammatory tumour microenvironment (TME) plays a crucial role in development and progression of malignant tumours^6,7^. The TME comprises stromal cells, blood vessels, activated fibroblasts, extracellular matrix as well as reactive oxygen species (ROS) producing innate and adaptive immune cells^8–10^. IL6, TGFβ and TNFα expressed in TME generates ROS triggering cell proliferation and survival^11^. ROS is also formed by NADPH oxidases, which can be activated by various growth factors^12,13^. ROS generated by oxidative cellular stress play an important role in signalling pathways through AKT and ERK1/2 and activation of hypoxia inducible factor-1 (HIF-1)^14,15^. Thioredoxin-interacting protein (TXNIP) contribute substantially to accumulation of intracellular ROS by inhibiting the antioxidative function of thioredoxin (TXN)^16^. TXNIP expression and elevated level of ROS is required for VEGF-mediated VEGFR2 activation and proliferation of endothelial cells during tumor angiogenesis^17,18^. Under oxydative stress TXNIP interacts with NLRP3 inflammasome leading to maturation and secretion of inflammatory cytokine IL1β^19,20^. It was shown that IL1β mediates epithelial to mesenchymal transition of proximal tubular cells of kidney^21^.

Controversial results have been published on TXNIP expression and tumour progression. High level of TXNIP expression was associated with a significantly shorter survival of patients with non-small cell lung cancer and invasive growth of hepatocellular carcinoma^22,23^. On the other hands, lack or reduced expression of TXNIP in cancer cell lines, experimental mouse models as well as in tumour tissues suggested that TXNIP is a tumour suppressor gene^24–30^. It has also been reported that decreased TXNIP RNA expression is associated with poor prognosis of patients with clear cell renal cell carcinoma^31^. To establish the role of TXNIP and downstream genes HIF1α and IL1β in the biology of cRCC, we have applied immunohistochemistry to tissue multi-arrays containing tumours of patients without detectable metastases at the time of operation.

## Results

### Expression of TXNIP in normal kidney

TXNIP showed a weak expression at the luminal surface of proximal tubular cells and a strong cytoplasmic expression in connecting and collecting duct cells in normal adult kidneys, whereas the loop of Henle and distal convoluted tubules were negative (data not shown). Vascular smooth muscle cells and endothelial cells of small kidney arteria displayed a weak TXNIP expression. We did not find nuclear TXNIP expression in normal adult kidney. No expression of HIF1α and IL1b has been seen in normal kidney tissue.

### Expression of TXNIP in conventional RCC

**Figure 1:**
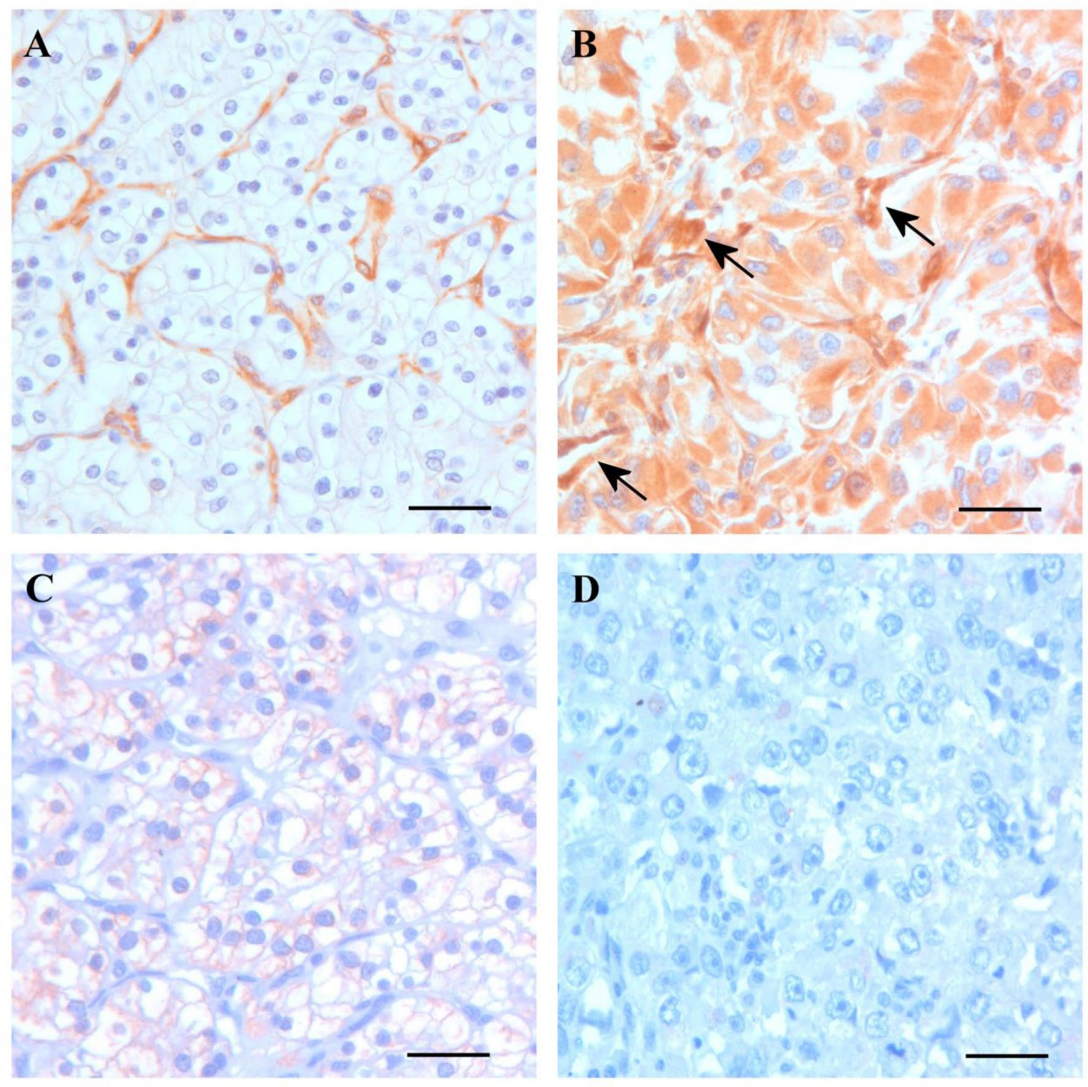
Expression of TXNIP and IL1β in the two groups of conventional RCC. (**A**) No cytoplasmic TXNIP expression was detected in the first group of tumours of excellent prognosis. Endothel cells of the fine vascular network display TXNIP positivity. (**B**) Tumour of the second group with poor prognosis display strong cytoplasmic TXNIP expression and contains few tourtous vessels (arrows). (**C**) Grade 1 tumour of the first group displaying cytoplasmic IL1β expression. (**D**) Grade 2 tumour of the second group without IL1β immunostaining. No IL1β expression can be seen in the tumour microenvironment. Scale bar: 40 μm.

Results of immunohistochemistry of 691 cRCC separated two groups of tumours. One group of 512 tumours (74%) without cytoplasmic expression of TXNIP (Fig. 1A), and the other group displaying medium or strong cytoplasmic TXNIP staining in 95 (14%) and 84 (12%) cases, respectively. (Fig. 1B). As the first Kaplan–Meier analysis revealed that patients with medium or strong cytoplasmic TXNIP expression have similar disease-free survival, we have evaluated the results of cytoplasmic TXNIP immunohistochemistry in correlation to clinical and pathological parameters as negative or positive.

### Expression of IL1β and HIF1α in conventional RCC

We used the same series of TMA as for TXNIP immunohistochemistry. A granular cytoplasmic IL1β expression has been seen in 601 of the 691 conventional RCCs (Fig. 1C), whereas 90 cases showed a negative immunreaction with IL1β antibody (Fig. 1D). We did not find HIF1α expression in any of the tumour cells analysed in this study. Positive HIF1α staining has been detected in some endothelial cells and in tumour infiltrating immune cells.

### TXNIP expression in tumour microvessels

**Figure 2:**
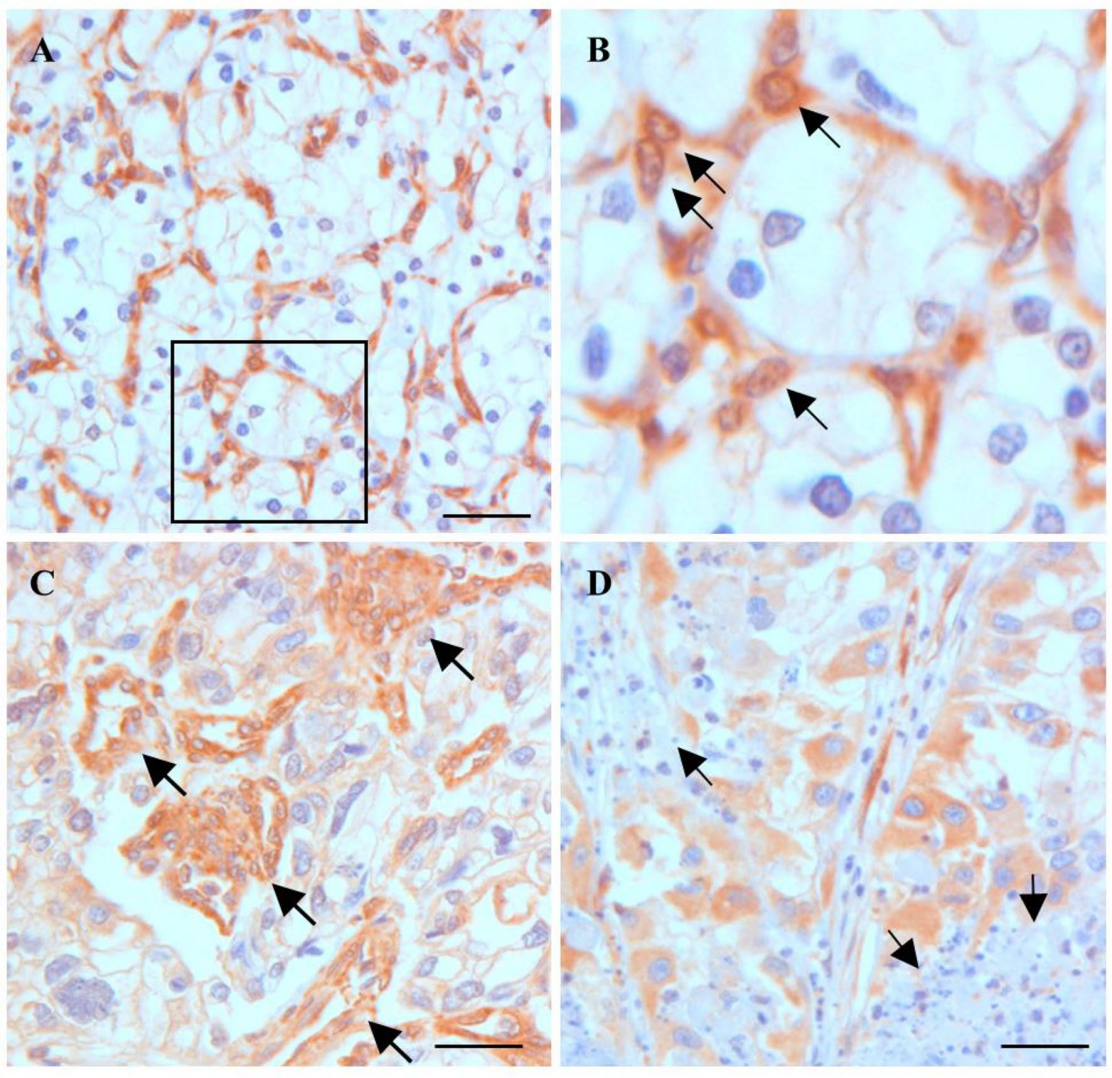
Expression of TXNIP in conventional RCC. (**A**) Strong TXNIP staining in endothelial cells but lack of expression in tumour cells. (**B**) Enlargement of the insert from A shows TXNIP translocation into endothel cell nuclei (arrows). (**C**) Irregular tortously growing TXNIP positive vessels in a grade 3 conventional RCC (arrows). (**D**) Small necrotic tumour areas are surrounded by tumour cells showing high expression of TXNIP protein (arrows). Scale bar: 40 μm.

Irrespectively of positive or negative cytoplasmic expression in tumour cells TXNIP showed a strong expression in endothelial cells of the tumour stroma. In highly differentiated cRCC resembling the classic “Grawitz” tumour a fine network of TXNIP positive endothel cells and tumour cells without TXNIP staining was seen (Fig. 2A). The overwhelming majority of endothelial cells displayed a nuclear TXNIP expression (Fig. 2B). In the second group of tumours with medium or high TXNIP expression in tumour cells only few vessels has been recognized. The majority of TXNIP positive tumours displayed unorganized, irregular or tortuous tumour vessels (Fig. 2C) as compared to those seen in well differentiated cRCC (Figs. 1A and 2A). Around small necrotic tumour areas tumour cells displayed strong cytoplasmic TXNIP immunoreaction (Fig. 2D). As vascular TXNIP expression has been detected in all tumours analysed in this cohort, we evaluated only the cytoplasmic TXNIP expression in relation of tumour the progression.

### Correlation analysis

**Figure 3:**
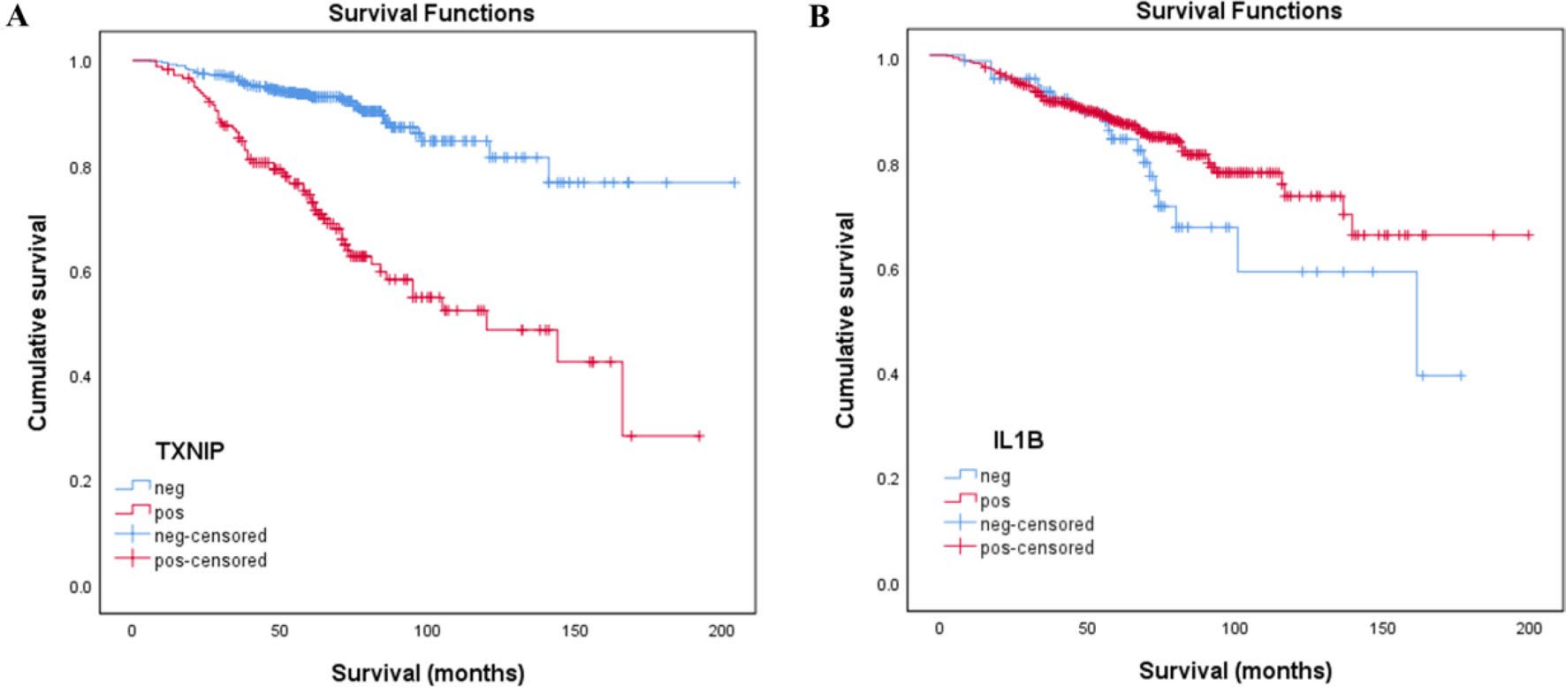
Kaplan–Meier estimates for disease free survival for 691 patients without metastatic disease at the time of operation. (**A**) Positive cytoplasmic immunostaining of TXNIP protein indicates its prognostic value (*p* < 0.001). (**B**) Although, cytoplasmic positivity of IL1β indicates a better outcome of the disease, it is not significant (*p* = 0.109).

The TXNIP expression in tumour cells was significantly correlated with tumour size, grade and T-classification and tumour necrosis as well as postoperative progression of cRCCs (Table 1, all *p* < 0.001). Kaplan–Meier analysis revealed that patients having a cRCC with cytoplasmic expression of TXNIP protein have a significantly shorter disease-free survival compared to those without TXNIP expression (Fig. 3A). Univariate Cox regression analysis revealed that tumor size, grade, T classification, necrosis as well as cytoplasmic TXNIP positivity were significantly associated with postoperative tumour progression (all *p* < 0.001). However, in multivariate Cox regression analysis only cytoplasmic TXNIP expression and necrosis remained independent predictor of cancer progression indicating two times higher risk of postoperative tumour relapse (Table 2). Kaplan–Meier analysis showed that patients having a cRCC without cytoplasmic expression of IL1β protein have a shorter disease-free survival compared to those with cytoplasmic IL1β expression (Fig. 3B). In multivariate cox regression analysis no correlation between IL1β expression and postoperative relapse has been found. IL1β protein level showed a significant correlation only with histological grade (*p* < 0.05).

**Table 1:** Association of TXNIP expression with clinical-pathological parameters of conventional RCCs without metastasis at the time of operation (n = 691).

	Nr of cases (691)	TXNIP expression		*p*-value
		Negative (512)	Positive (179)	
**Gender**				0.001
Male	406	282	124	
Female	285	230	55	
**Status**				< 0.001
AWD	579	465	114	
PTR	112	47	65	
**Size**				0.001
< 4 cm	272	220	52	
4 < x < 7 cm	269	196	73	
> 7 cm	150	96	54	
**T Stadium**				< 0.001
pT1	511	401	110	
pT2	94	66	28	
pT3	86	45	41	
**Grade**				< 0.001
G1	456	382	74	
G2	180	114	66	
G3	55	16	39	
**Necrosis**				< 0.001
No	608	479	129	
Yes	83	33	50	
**Stage**				< 0.001
I	504	397	107	
II	167	104	63	
III	20	11	9	

*AWD* alive without disease, *PTR* postoperative tumour relapse.

**Table 2:** Multivariate analysis of positive TXNIP expression in 691 conventional RCCs.

	RR	95.0% CI for Exp(B)		*p*-value
		Lower	Upper	
**Gender**				
Male/female	0.880	0.587	1.321	0.538
**Size (cm)**				
< 4				0.178
4 < x < 7	1.246	0.636	2.441	0.521
> 7	0.767	0.334	1.758	0.531
**T Stadium**				
pT1				0.002
pT2	2.553	0.304	21.457	0.388
pT3	5.976	0.768	46.480	0.088
**Grade**				
G1				0.062
G2	1.655	0.982	2.788	0.059
G3	2.102	1.120	3.944	0.021
**Necrosis**				
No/yes	1.928	1.241	2.997	0.004
**Stage**				
I				0.884
II	1.680	0.212	13.334	0.624
III	1.654	0.219	12.486	0.626
**TXNIP**				
Cytoplasmic neg/pos	2.034	1.334	3.102	0.001

## Discussion

We analysed the expression of TXNIP protein in a large cohort of cRCC without detectable metastasis at the time of operation. The Kaplan–Meier survival analysis indicated that patients with tumours dysplaying cytoplasmic TXNIP protein expression have significantly shorter tumour free survival (*p* < 0.001). Multivariate analysis revealed that these patients have a more than two times higher risk to develop a metastatic disease during the median follow-up of 73 months (*p* = 0.001).

The only report on TXNIP and renal cancer evaluated RNA expression data deposited in Cancer Genome Atlas (TCGA) and concluded that decreased expression of TXNIP predicts a poor prognosis^31^. Reduced expression or lack of expression of TXNIP was associated with the development of oxidative stress induced experimental RCC in rat^29^. Decrease of TXNIP RNA in bladder cancer and development of bladder cancer in TXNIP-KO mice has been described^27^. Low expression of TXNIP was observed in high grade glioma tissues by comparing to low grade tumours^25^. In experimental mouse model the silencing of TXNIP increased the predisposition to hepatocellular carcinoma (HCC)^30^. In two studies expression of TXNIP was detected in all of primary human HCC, but the expression was decreased in 66% and 50% of the cases, respectively^28,32^. Based on these data TXNIP was suggested to be a tumour suppressor gene. However, in line with our results a significantly increased expression of TXNIP and elevated ROS level was associated with invasive growth in human hepatocellular carcinoma and high expression of TXNIP was an independent prognostic factor in non-small lung cancer^22,23^.

TXNIP is a member of α-arrestin protein family and by directly binding to the antioxidant TXN blocks its reducing potential^13^. TRXs are small redox active proteins that play an important role to maintain the cellular redox balance under normal conditions. Elevated TXNIP expression and oxidative stress influence several biological functions, regulates cell growth, differentiation and apoptosis^33,34^. By supporting the ROS production TXNIP promotes endothelial cells proliferation and angiogenesis by activating the transcription factor NF-kB and regulating the vascular endothelial growth factor (VEGF), and vascular endothelial growth factor receptor 2 (VEGFR2) signalling^18,35,36^. The importance of TXNIP in the maintenance of endothelial homeostasis was recently demonstrated by a TXNIP-KO mouse experiment^37^. ROS are also involved in the induction of HIF family transcription factors the major signalling components downstream of hypoxia^38^. Many of the HIF target proteins are also involved in the angiogenesis. In our study no HIF1α expression has been observed the tumours cells.

We showed in this study that slowly growing, differentiated cRCC with excellent clinical outcome displays an organized vascular network consisting of TXNIP positive endothelial cells. The translocation of TXNIP into the nuclei of endothelial cells of tumour supporting vessels indicates an increased level of ROS. It was shown previously that forced expression of TXNIP in isolated microvascular endothelial cells results in its nuclear translocation and activates the NF-kB pathway leading to expression of pro-inflammatory cytokines including IL1β^20,39,40^. Based on our results that IL1β expressed preferentially in a group of tumours showing strong TXNIP nuclear expression in supporting endothelial cells indicates that the IL1β expression is mediated through the NFkB pathway, rather than direct activation of NLRP3 inflammasome. Recently, it was shown that IL1β mediates epithelial to mesenchymal transition of proximal tubular cells of kidney^21^. Conventional RCC derives from proximal tubular cells of the kidney. However, we found IL1β expression in the group of cRCC, with excellent outcome of disease and not in the group of tumours with increased cell motility leading to high risk of postoperative metastatic growth.

We showed in this study that rapid growth of tumour cells in highly malignant cRCC with metastatic capacity exceeded the proliferation of oxygen supplying capillaries. The tumour cell/blood vessel relation is strongly shifted in favour of tumour cells and lead to inefficient vascularisation by unorganized and tortuous vessels. Elevated ROS level can impair the function of important proteins and interfere with metabolic pathways. In hypoxemic microenvironment the ATP is mostly produced via high rate of anaerobic glycolysis and the Warburg effect leads to lactic acid fermentation and further increases oxidative stress^41,42^. The hypoxemic and acidic TME may dramatically change the oxidative-reductive balance by producing high level of ROS which in increased concentration may inhibit tumour growth^43–45^. The high level of oxidative stress may cause serious damage of tumour DNA and proteins leading to death of cancer cells^46^. We showed in this study that the highest expression of TXNIP protein occurred in tumour areas with small circumscribed tumour cell necrosis. In multivariate analysis, in addition to TXNIP positivity, tumour necrosis was presented as independent prognostic factor of postoperative tumour relapse (*p* = 0.004).

In conclusion, our current study demonstrates that cRCC displaying a fine organised capillary network with nuclear translocation of TXNIP and expressing IL1β have a good prognosis. In contrary, we showed a significant correlation between cytoplasmic TXNIP expression, inefficient vascularisation by unorganized and tortuous vessels causing tumour cell necrosis and postoperative tumour relapse of cRCC.

## Material and methods

### Patients

We have analysed a cohort of cRCCs obtained from 691 patients operated between 2000 and 2013 without clinically detectable metastasis at the first observation described previously^47^. Data on regular follow-up and tumour specific death was obtained from Tumour Registry of the Department of Urology in accordance with the relevant institutional guidelines and regulations. Follow-up was defined as a time from the operation until the last recorded control or cancer specific death. The pertinent clinical and pathological data in are presented in Table 1. Of the 691 patients, 406 (59%) were males and 285 (41%) females, the mean age of the cohort was 61.3 ± 11.2 years (range 23–88 years). The average tumour size was 50.2 ± 25.8 mm. During the median follow-up of 73 ± 28 months, tumour relapse was observed in 112 patients (16%). Of 691 tumours, 511 (74%) were classified as pT1 including 308 (45%) pT1a tumour. The overwhelming majority of cRCCs (456 of 691) displayed G1 tumour grade. Regarding to the tumour stage, 671 (97%) of tumours were designed to stage I and II.

### Tumour samples and tissue microarray (TMA)

The histological diagnosis and TNM classification was established by a genitourinary pathologist (GK) according to the Heidelberg and TNM classification and by applying 3 trier grading^48,49^. The collection and use of all tissue samples for this study was approved by the Ethics Committee of the University Pecs, Hungary (No. 5343/2014). All participants sign an informed consent that after establishing the histological diagnosis the rest of formalin fixed and paraffin embedded material can be used for immunohistochemistry. We have identified representative tumour areas on haematoxylin and eosin stained slides and the corresponding paraffin blocks were used for TMA construction. Three to five core biopsies with a diameter of 0.6 mm were taken from each tumour and were placed in the recipient block using a Manual Tissue Arrayer (MTA1, Beecher Instruments, Inc., Sun Prairie, USA). We have also analysed foetal and adult kidneys for establishing the TXNIP, HIF1α and IL1β expression in normal renal tissue.

### Immunohistochemistry

After removing the paraffin and rehydration the 4 μm thick sections were subjected to heat-induced epitope retrieval in citrate buffer, pH 6.0 for IL1β and EnVision FLEX Target Retrieval Solution, high pH (DAKO, Glostrup, Denmark) for TXNIP and HIF1α in 2100-Retriever (Pick-Cell Laboratories, Amsterdam, The Netherlands). Endogenous peroxidase was blocked with Envision FLEX Peroxydase Blocking Reagent (DAKO) for 10 min at room temperature. Slides were then incubated at room temperature for one hour with monoclonal rabbit anti-TXNIP antibody (EPR14774, ab 188865, abcam, Cambridge, UK) at the dilution of 1:200; monoclonal mouse interleukin 1 beta antibody (AM06692SU-N, Origene Rockville, MD, USA) at the dilution of 1:200; and monoclonal rabbit anti HIF-1 alpha antibody (EP1215Y, abcam, UK) at the dilution of 1:600. EnVision FLEX horse-radish-peroxydase conjugated secondary antibody (DAKO) was applied for 30 min at room temperature and colour was developed using the DAB substrate (DAKO). Tissue sections were counterstained with Mayer's haematoxylin (Lillie’s modification, DAKO) and after 10 s bluing were mounted with Pertex. For negative control the primary antibody was omitted and for positive control we used the normal kidney biopsies included into the TMAs. We have scored the TXNIP staining as negative, medium and high expression. If at least one of the 3 or more core biopsies was positive, we evaluated the tumour as positive.

### Statistical analysis

Statistical analysis was carried out as described earlier^47^. Correlations between categorical variables were estimated with Fisher’s exact test. Estimates of the cumulative survival distributions were calculated by the Kaplan–Meier method, and the differences between the groups were compared using the log-rank test. The significance of clinical-pathological variables was evaluated using the univariate and multivariate Cox proportional hazard regression model. Analysis was performed using IBM SPSS Statistics v.27 for Windows (Inc. Chicago IL, USA). *p*-value < 0.05 was considered the limit of statistical significance.

## Data Availability

The datasets are available from the corresponding authors on reasonable request.
